# Parental Diabetes: The Akita Mouse as a Model of the Effects of Maternal and Paternal Hyperglycemia in Wildtype Offspring

**DOI:** 10.1371/journal.pone.0050210

**Published:** 2012-11-28

**Authors:** Corinna Grasemann, Maureen J. Devlin, Paulina A. Rzeczkowska, Ralf Herrmann, Bernhard Horsthemke, Berthold P. Hauffa, Marc Grynpas, Christina Alm, Mary L. Bouxsein, Mark R. Palmert

**Affiliations:** 1 Kinderklinik II, Division of Paediatric Endocrinology, UK- Essen and The University of Duisburg-Essen, Essen, Germany; 2 Division of Endocrinology and Department of Paediatrics, Hospital for Sick Children, The University of Toronto, Toronto, Canada; 3 Beth Israel Deaconess Medical Center, Department of Orthopedic Surgery, Harvard University, Boston, Massachusetts, United States of America; 4 Kinderklinik I, Division of Neonatology, UK- Essen and The University of Duisburg-Essen, Essen, Germany; 5 Department of Human Genetics, UK- Essen and The University of Duisburg-Essen, Essen, Germany; 6 Samuel Lunenfeld Research Institute and The University of Toronto, Toronto, Canada; 7 Division of Endocrinology and Department of Physiology, Hospital for Sick Children, The University of Toronto, Toronto, Canada; Ohio State University Medical Center, United States of America

## Abstract

**Aim/Hypothesis:**

Maternal diabetes and high-fat feeding during pregnancy have been linked to later life outcomes in offspring. To investigate the effects of both maternal and paternal hyperglycemia on offspring phenotypes, we utilized an autosomal dominant mouse model of diabetes (hypoinsulinemic hyperglycemia in Akita mice). We determined metabolic and skeletal phenotypes in wildtype offspring of Akita mothers and fathers.

**Results:**

Both maternal and paternal diabetes resulted in phenotypic changes in wildtype offspring. Phenotypic changes were more pronounced in male offspring than in female offspring. Maternal hyperglycemia resulted in metabolic and skeletal phenotypes in male wildtype offspring. Decreased bodyweight and impaired glucose tolerance were observed as were reduced whole body bone mineral density and reduced trabecular bone mass.

Phenotypic changes in offspring of diabetic fathers differed in effect size from changes in offspring of diabetic mothers. Male wildtype offspring developed a milder metabolic phenotype, but a more severe skeletal phenotype. Female wildtype offspring of diabetic fathers were least affected.

**Conclusions:**

Both maternal and paternal diabetes led to the development of metabolic and skeletal changes in wildtype offspring, with a greater effect of maternal diabetes on metabolic parameters and of paternal diabetes on skeletal development. The observed changes are unlikely to derive from Mendelian inheritance, since the investigated offspring did not inherit the Akita mutation. While fetal programming may explain the phenotypic changes in offspring exposed to maternal diabetes *in-utero*, the mechanism underlying the effect of paternal diabetes on wildtype offspring is unclear.

## Introduction

In data from a human cohort from the early 20^th^ century, Barker observed that infants with the lowest birth weight had the highest risk of developing cardiovascular disease. This observation formed the basis for the hypothesis that an adverse *in-utero* environment could have a programming effect on the developing fetus, resulting in lifelong effects [1].

The impact of *in-utero* energy restriction on the development of so-called ‘later in life diseases’, such as obesity, diabetes and cardiovascular disease, has been recognized and confirmed in subsequent epidemiological studies. Surprisingly, not only fetal growth restriction, but also fetal overnutrition, seems to pose a risk for the development of obesity and an unfavorable metabolic profile [2].

Specifically, offspring of diabetic mothers seem to carry a risk for the development of obesity and diabetes, as shown in population studies in Pima Indians, American youth with Type 2 Diabetes, and children of mothers affected by gestational diabetes [2], [3].

While part of this risk is likely due to the transmission of DNA sequence variants (Mendelian inheritance), fetal programming appears to be an additional factor. Whether this is caused by the maternal hyperglycemia alone or mediated by other, diabetes-related change, in the maternal organism remains unclear. Recent data suggest a programming effect by higher glucose concentration during pregnancy [4].

To date, limited animal studies that model maternal diabetes have been used to investigate this phenomenon further. In genetic mouse models of spontaneous gestational diabetes [5] and in a rat model of hyperglycemia induced during pregnancy by continuous glucose infusion [6], obesity and metabolic changes have been observed in offspring. Artificial hyperinsulinemia in newborn rats results in obesity, hyperglycemia and hyperinsulinemia in adulthood [7]. Since these models investigate the effects of hyperinsulinemic diabetes (Type 2 diabetes and gestational diabetes) on offspring, they do not allow assessment of effects of high glucose levels independent of high insulin concentrations in pregnancy.

So far, only streptozotocin induced diabetes models have been used to model the effects of maternal type 1 diabetes and MODY on offspring [6] [8]. Thus, there is a lack of genetic models for maternal hypoinsulinemic diabetes and its effects on offspring.

The initial research about the developmental origins of adult disease focused on risks related to the maternal environment. However, recent work has shown that not only maternal, but also paternal environment has an effect on offspring in animal models [9], suggesting the need to investigate the effects of both maternal and paternal diabetes on offspring.

We hypothesized that both maternal and paternal hypoinsulinemic hyperglycemia would exert later life effects on offspring and, therefore, sought to develop a mouse model that could be used to investigate these effects more fully. Here we report the results of breeding wildtype mice with heterozygous Akita mice, a strain that develops hypoinsulinemic hyperglycemia in the heterozygote state due to an *Ins2^C96Y^* mutation, and phenotyping the wildtype offspring through assessment of metabolic status, body composition and skeletal development. Our results suggest that the Akita mouse is likely to be a useful model for investigating the phenotypic spectrum and bases of maternal and paternal diabetes effects on offspring.

## Results

**Figure 1:**
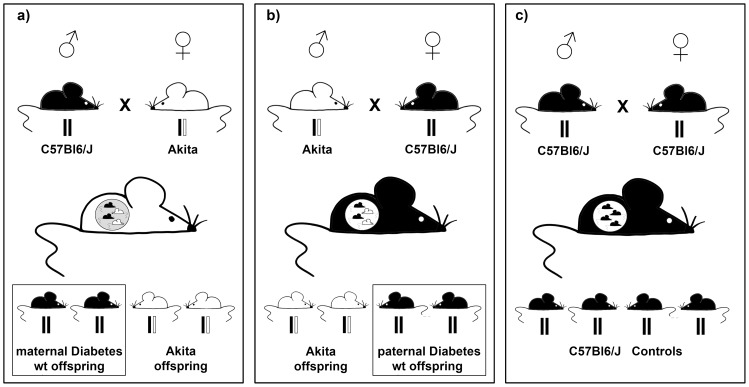
Schematic of Breeding Strategy. Generation of wildtype C57Bl6/J offspring of **a**) diabetic, heterozygous Akita females, **b**) diabetic, heterozygous Akita males and **c**) wildtype mice (control offspring). Offspring of Akita mothers will have been exposed to *in-utero* hyperglycemia. All offspring of Akita parents were genotyped and only the wildtype offspring were assessed subsequently.

For effects of **maternal** diabetes, heterozygous Akita females were bred with wildtype C57Bl6/J males and wildtype offspring were assessed. For effects of **paternal** hyperglycemia, heterozygous Akita males were bred with wildtype C57Bl6/J females, again wildtype offspring were assessed. Offspring of wildtype C57Bl6/J breeders, unrelated to the heterozygous Akita breeders, served as controls. For schematic representation of breeding strategy see Figure 1.

**Figure 2:**
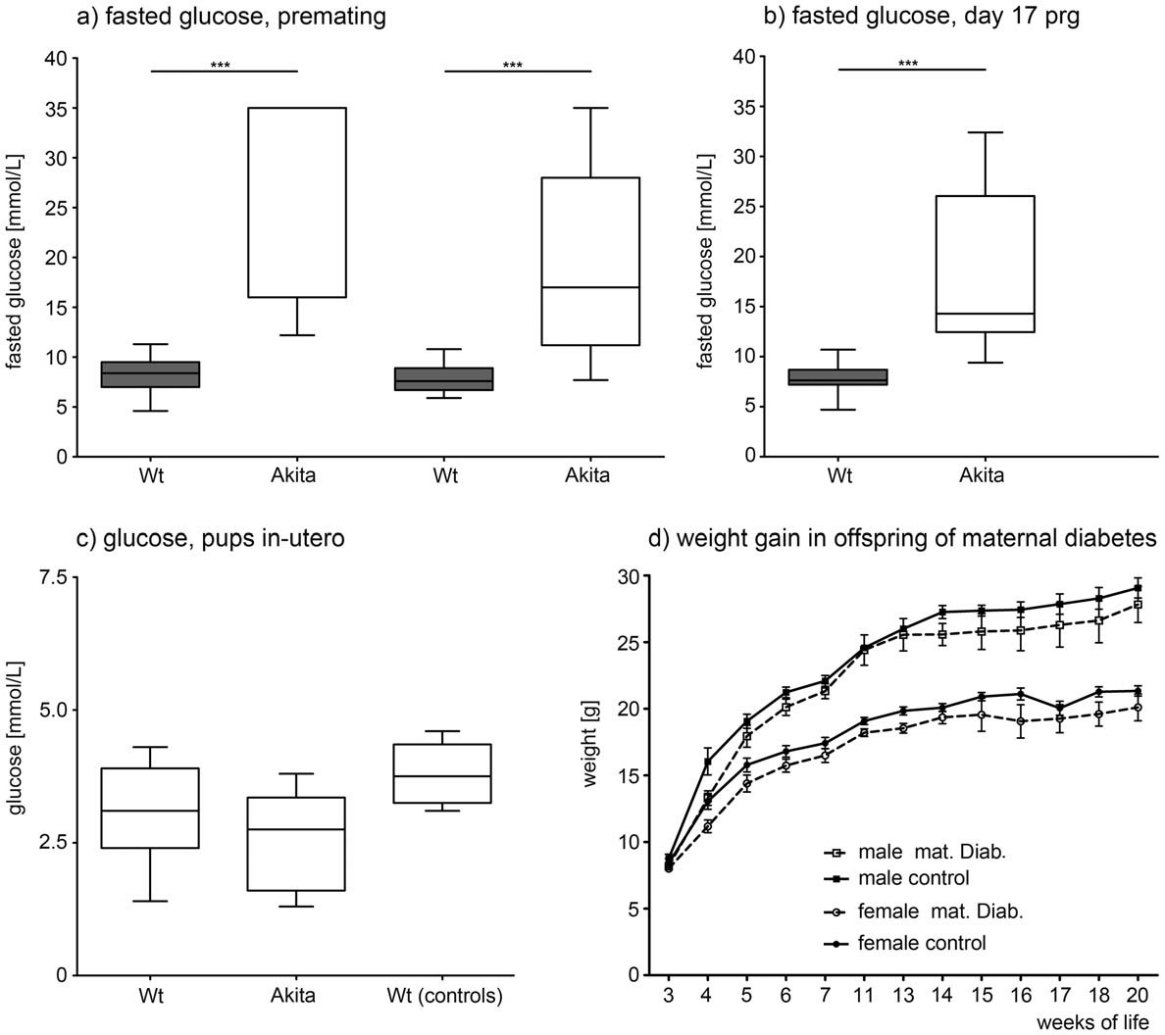
Premating and Pregnancy Glucose Values. Boxplots showing the smallest observation (lower bar), lower and upper quartile (box), median (line in the box) and largest observation (upper bar) of fasted serum glucose in mmol/L in **a**) WT and Akita males (n = 26 WT, 8 Akita) and females (n = 22 WT, 19 Akita) prior to mating, **b**) WT and Akita females on day 17 of pregnancy and **c**) wildtype (n = 10) and heterozygous (Akita) pups (n = 7) *in-utero* on day 17 of pregnancy and WT pups (n = 5) from control breedings on day 17 of pregnancy. *** indicate p<0.001 **d**) Weight gain from weaning to 20 weeks of life for male and female offspring of maternal diabetes and controls (mean weight in g ± SEM, n 8–15 per group). Offspring of diabetic mothers weighed less from weaning (for males: 8.16+0.9 g vs. 8.75+0.8 g in controls and for females: 8.0+0.6 g vs 8.4+1.0 g in controls) to sacrifice.

Fasting glucose levels in heterozygous Akita males (31.2±7.6 mmol/L) and females (19.2±8.7 mmol/L) were elevated compared to wildtype breeders premating (8.2±1.9 and 7.8±1.4 mmol/L, respectively) and during pregnancy.(Figure 2A and 2B). Glucose levels of heterozygous and wildtype fetuses did not differ on day 17 of pregnancy (Figure 2C).

Phenotypes of **wildtype** offspring of Akita matings and control matings were measured and analysed at 14 and 26 weeks. Phenotypic data of all animals studied were included for the multivariate three-way ANOVA analysis. Results (p values) are displayed in Table 1.

**Table 1:** Results 3-way ANOVA.

					Interaction of			
	Unit	Group	Age	Sex	Group*Age	Group*Sex	Age*Sex	Group*Age*Sex
**Weight**	g	**0.001**	**0.001**	**0.001**	0.82	**0.001**	**0.02**	**0.015**
**Length**	cm	**0.001**	**0.001**	**0.001**	**0.007**	**0.045**	**0.001**	**0.019**
**Glucose**	mmol/l	**0.001**	0.24	0.09	0.122	0.44	**0.003**	0.45
**AUC**		**0.001**	0.288	**0.001**	0.396	**0.035**	0.445	0.07
**Insulin**	ng/ml	0.409	0.933	**0.018**	0.783	0.555	0.642	0.899
**Leptin**	ng/ml	**0.007**	0.73	**0.009**	0.061	0.101	0.39	0.653
**IGF-1**	ng/ml	0.779	0.628	**0.001**	na	na	0.502	na
**BMD**	g/cm^2^	**0.001**	**0.001**	**0.002**	0.164	0.164	0.151	0.636
**BMC**	g	**0.001**	**0.001**	**0.007**	0.245	0.689	0.673	0.795
**Area**	cm^2^	**0.002**	0.123	0.143	0.331	0.91	0.68	0.508
**LBW**	g	**0.004**	**0.001**	**0.001**	0.119	**0.003**	0.587	0.807
**BFAT**	g	**0.012**	**0.0012**	**0.004**	0.159	0.423	0.298	0.6339
**%Fat**	%	0.088	0.413	0.133	0.476	0.94	0.164	0.558

P-values for influence of group (maternal diabetes/paternal diabetes/control), age and sex on phenotypic markers are provided, as well as p-values for interactions of group and age, age and sex, sex and group, sex and age are given. Cells with p<0.05 are highlighted.

The ANOVA analysis demonstrates that **group**, in form of maternal or paternal diabetes as well as **age** and **sex** of offspring all affect phenotype. There is a significant effect (p<0.05) of maternal or paternal diabetes (**group**) on most metabolic markers studied in offspring, including: weight, length, fasted glucose, glucose clearance after ip glucose challenge (AUC), and leptin. (Table 1)

Both maternal and paternal diabetes also significantly influence bone mineral properties including WBBMD, WBBMC and Bone Area as measured by pDXA. Lean body weight (LBW) and Body Fat (BFat), were also affected. (Table 1)

**Age** at phenotyping (14 or 26 weeks) influences weight, length, WBBMD, WBBMC, LBW and BFat, and **offspring sex** influences almost all markers studied, including weight, length, AUC, insulin, leptin, IGF-1, WBBMD, WBBMC, LBW and BFat, indicating a strong sexual dimorphism. (Table 1)

Further statistical analysis was then performed analyzing effects of maternal and paternal diabetes separately for each sex and age group. Results of these sub analyses are discussed below. Due to space constraints and for ease of presentation, the main metabolic and skeletal findings are also displayed in Table 2 for male and Table 3 for female offspring. For the reader interested in other outcomes, results of all investigated metabolic and skeletal markers are displayed in Table S1.

**Table 2:** Phenotypic results for metabolic and skeletal markers in male offspring separated by age and group.

			Maternal Diabetes			Paternal Diabetes			Controls		
		Unit	Mean	SD	n (Prg)	Mean	SD	n (Prg)	Mean	SD	n (Prg)
**14 weeks**	**Weight**	g	**25.45** *	**2.37**	**35 (16)**	**26.02** *	**1.65**	**29 (13)**	27.6	1.74	62 (23)
	**Length**	cm	9.75	0.21	16 (10)	**10.1** *	**0.2**	**5 (3)**	9.81	0.17	20 (12)
	**Glucose**	mmol/L	**9.64** *	**1.67**	**33 (16)**	8.6	1.5	26 (13)	8.52	1.69	53 (20)
	**AUC**		**2279** *	**672.7**	**32 (16)**	1984.13	514.9	23 (12)	1692.8	527.6	47 (19)
	**Insulin**	ng/ml	0.98	0.31	13 (9)	0.98	0.41	7 (5)	1.23	0.56	17 (6)
	**Leptin**	ng/ml	1.14	0.65	9 (4)	1.66	1.78	6 (4)	2.4	1.8	10 (5)
	**BMD**	g/cm^2^	**0.050** *	**0.003**	**16 (10)**	**0.049** *	**0.0028**	**5 (3)**	0.053	0.0019	20 (12)
	**BMC**	g	**0.43** *	**0.052**	**16 (10)**	0.42	0.039	5 (3)	0.48	0.037	20 (12)
	**Area**	cm^2^	**8.45** *	**0.55**	**16 (16)**	8.66	0.353	5 (3)	9.04	0.49	20 (12)
	**Tb.Th**	mm	0.057	0.007	8 (5)	**0.052** *	**0.006**	**7 (4)**	0.058	0.006	17 (10)
	**Tb.N**	/mm	4.96	0.36	8 (4)	**4.72** *	**0.15**	**7 (4)**	5.1	0.39	17 (10)
	**Cort. area**	mm^2^	0.808	0.123	8 (4)	**0.749** *	**0.071**	**8 (4)**	0.818	0.083	17 (10)
	**BA/TA**	%	43.5	1.7	8 (4)	41.5	1.6	8 (4)	43	2.5	17 (10)
	**Cort. Th.**	mm	0.178	0.012	8 (4)	**0.168** *	**0.009**	**8 (4)**	0.177	0.01	17 (10)
**26 weeks**	**Weight**	g	30.4	1.9	18 (6)	**29.8** *	**1.8**	**18 (6)**	31.9	2.56	38 (14)
	**Length**	cm	10.1	0.58	7 (4)	10.25	0.26	10 (5)	10.2	0.29	20 (9)
	**Glucose**	mmol/L	9.01*	**1.74**	**17 (6)**	**9.03** *	**2.19**	**18 (6)**	7.4	1.66	38 (14)
	**AUC**		2425*	**797**	**15 (5)**	1799.2	512	18 (6)	1575	418	37 (14)
	**Insulin**	ng/ml	0.76	0.24	8 (4)	1.1	0.54	6 (2)	1.23	0.87	13 (5)
	**Leptin**	ng/ml	1.78	0.38	6 (3)	**0.56** *	**0.43**	**4 (2)**	3.63	2.2	13 (5)
	**BMD**	g/cm^2^	0.053	0.003	7 (4)	0.055	0.003	10 (5)	0.056	0.0027	20 (9)
	**BMC**	g	0.48	0.06	7 (4)	0.48	0.044	10 (5)	0.51	0.039	20 (9)
	**Area**	cm^2^	9.05	0.6	7 (4)	8.81	0.78	10 (5)	9.16	0.48	20 (9)
	**LBW**	g	**22.8** *	**1.6**	**7 (4)**	23.7	1.6	10 (5)	24.99	1.84	20 (9)
	**Tb.Th**	Mm	**0.058** *	**0.006**	**8 (5)**	**0.059** *	**0.006**	**7 (6)**	0.065	0.004	18 (8)
	**Tb.N**	/mm	4.23	0.16	8 (5)	4.1	0.32	7 (6)	4.34	0.34	18 (8)
	**Cort. area**	Mm^2^	0.84	0.088	8 (5)	0.794	0.052	7 (6)	0.877	0.064	18 (8)
	**BA/TA**	%	43.7	3.7	8 (5)	39.6	3	7 (6)	42.3	3.1	18 (8)
	**Cort. Th.**	mm	0.178	0.01	8 (5)	**0.164** *	**0.008**	**7 (6)**	0.179	0.011	18 (8)

Mean+SD and animal numbers (n) as well as numbers of pregnancies () are provided for male offspring at 14 and 26 weeks of age for phenotypic markers obtained in offspring of maternal diabetes, paternal diabetes and controls. Significant differences to control offspring (p<0.05) are highlighted in grey shaded boxes (*, p<0.05).

AUC: Area under the curve for glucose response after ipGTT, BMD: bone mineral density, BMC: bone mineral content, LBW: lean body weight, BFAT: body fat, Tb.Th.: trabecular thickness, Tb.N.: number of trabeculae, Cort. Area: Cortical area, BA/TA: Bone area/trabecular area, Cort. Th.: Cortical thickness.

**Table 3:** Phenotypic results for metabolic and skeletal markers in female offspring separated by age and group.

			Maternal Diabetes			Paternal Diabetes			Controls		
		Unit	Mean	SD	n (Prg)	Mean	SD	n (Prg)	Mean	SD	n (Prg)
**14 weeks**	**Weight**	g	19.22 *	**0.97**	**18 (10)**	20.48	1.42	27 (14)	20.69	1	42 (20)
	**Length**	cm	9.4	0.3	3 (3)	9.31	0.21	13 (6)	9.15	0.27	16 (7)
	**Glucose**	mmol/L	8.46	1.65	14 (9)	7.46	0.95	6 (4)	7.46	1.33	36 (18)
	**AUC**		1932.2 *	**787.1**	**14 (9)**	1700.1	688.8	6 (4)	1405.5	458.9	35 (18)
	**Insulin**	ng/ml	0.43	0.19	8 (4)	na	na		0.42	0.64	8 (3)
	**Leptin**	ng/ml	1.26	0.24	8 (4)	na	na		0.77	0.27	7 (3)
	**BMD**	g/cm^2^	0.046	0.001	3 (3)	0.048	0.002	5 (4)	0.049	0.002	16 (7)
	**BMC**	g	0.39	0.031	3 (3)	0.4	0.03	5 (4)	0.44	0.053	16 (7)
	**Area**	cm^2^	8.42	0.39	3 (3)	8.37	0.3	5 (4)	8.97	0.77	16 (7)
	**LBW**	g	16.27	1.3	3 (3)	16.74	1.36	5 (4)	15.94	1.19	16 (7)
	**Tb.Th**	mm	0.05	0.005	8 (4)	0.047	0.004	6 (4)	0.05	0.005	19 (7)
	**Tb.N**	/mm	3.41	0.21	8 (4)	3.38	0.5	6 (4)	3.5	0.27	19 (7)
	**Cort. area**	mm^2^	0.68	0.05	8 (4)	0.699	0.101	6 (4)	0.702	0.039	20 (7)
	**BA/TA**	%	43.5	2.4	8 (4)	44.2	2.9	6 (4)	44.5	1.4	20 (7)
	**Cort. Th.**	mm	0.169	0.011	8 (4)	0.173	0.019	6 (4)	0.175	0.007	20 (7)
**26 weeks**	**Weight**	g	21.05 *	**1.35**	**7 (4)**	24.98	2.11	15 (6)	24.16	1.63	28 (11)
	**Length**	cm	9.48	0.21	5 (3)	10.13 *	**0.23**	**9 (3)**	9.73	0.28	20 (11)
	**Glucose**	mmol/L	10.0 *	**1.16**	**6 (4)**	8.43	1.22	12 (6)	7.97	1.77	24 (10)
	**AUC**		1614.5	210.09	6 (4)	1370.7	325.33	5 (4)	1574.7	437.31	24 (10)
	**Insulin**	ng/ml	0.39	0.07	5 (3)	na	na		0.52	0.16	12 (7)
	**Leptin**	ng/ml	0.94	0.06	5 (3)	na	na		1.66	0.91	10 (6)
	**BMD**	g/cm^2^	0.052 *	**0.001**	**7 (5)**	0.055	0.002	9 (3)	0.055	0.001	20 (11)
	**BMC**	g	0.46	0.03	7 (5)	0.47	0.03	9 (3)	0.48	0.02	20 (11)
	**Area**	cm^2^	8.95	0.54	7 (5)	8.58	0.44	9 (3)	8.84	0.41	20 (11)
	**LBW**	g	16.84 *	**1.15**	**7 (5)**	19.96 *	**1.06**	**9 (3)**	18.66	1.37	20 (11)
	**BFAT**	g	**3.71**	**0.35**	**7 (5)**	5.62	1.24	9 (3)	5.19	1.01	20 (11)
	**%Fat**		**18.05**	**1.45**	**7 (5)**	21.85	3.67	9 (3)	21.64	2.98	20 (11)
	**Tb.Th**	mm	0.048	0.006	7 (5)	0.05	0.006	9 (3)	0.05	0.005	18 (10)
	**Tb.N**	/mm	2.76	0.14	7 (5)	2.61	0.3	9 (3)	2.66	0.19	18 (10)
	**Cort. area**	mm^2^	0.761 *	**0.043**	**7 (5)**	0.84	0.033	9 (3)	0.836	0.044	18 (10)
	**BA/TA**	%	47.6 *	**2**	**7 (5)**	48.8	1.3	9 (3)	49.2	1.8	18 (10)
	**Cort. Th.**	mm	0.192 *	**0.009**	**7 (5)**	0.203	0.006	9 (3)	0.203	0.007	18 (10)

Mean+STD and animal numbers (n) as well as numbers of pregnancies () are provided for male offspring at 14 and 26 weeks of age for phenotypic markers obtained in offspring of maternal diabetes, paternal diabetes and controls. Significant differences to control offspring (p<0.05) are highlighted in grey shaded boxes (*, p<0.05).

AUC: Area under the curve for glucose response after ipGTT, BMD: bone mineral density, BMC: bone mineral content, LBW: lean body weight, BFAT: body fat, Tb.Th.: trabecular thickness, Tb.N.: number of trabeculae, Cort. Area: Cortical area, BA/TA: Bone area/trabecular area, Cort. Th.: Cortical thickness.

### Effects of maternal diabetes on male offspring

#### Male offspring

**Figure 3:**
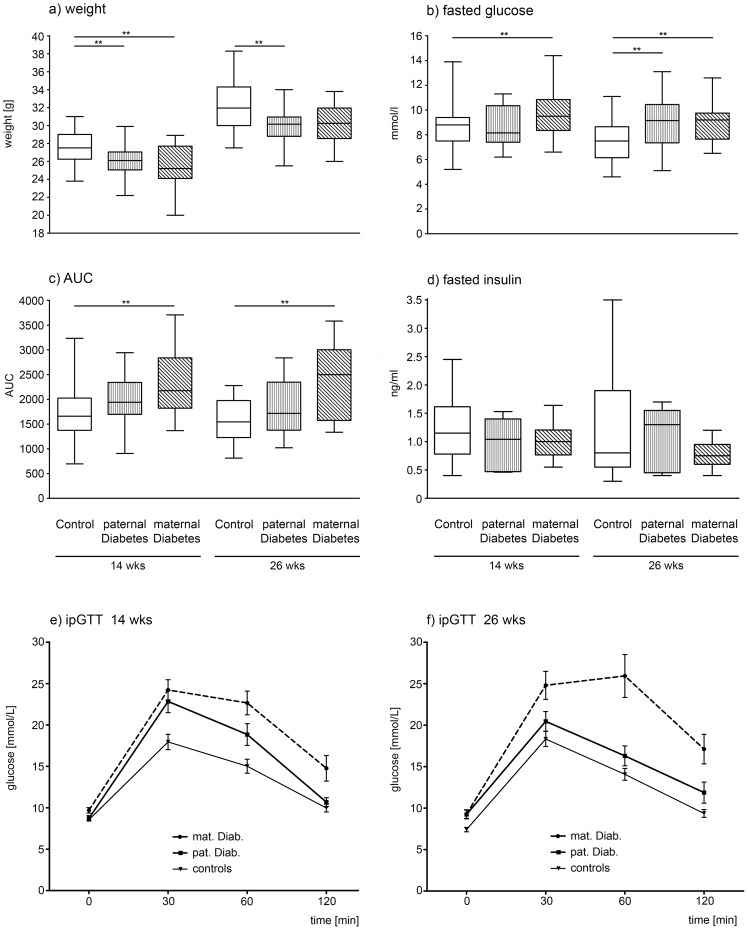
Metabolic Outcomes in Wildtype Male Offspring. Boxplots showing the smallest observation (lower bar), lower and upper quartile (box), median (line in the box) and largest observation (upper bar) of selected phenotypic markers: **a**) weight in g, **b**) fasted, glucose in mmol/L, **c**) AUC and **d**) fasted insulin (ng/ml) are given for male offspring of control breedings (open bars), paternal diabetes (longitudinal shading) and maternal diabetes (diagonal shading), at 14 and 26 weeks of age. Mean glucose ± STD during ipGTTs for offspring of maternal diabetes, paternal diabetes and controls are shown for 14 (**panel e**) and 26 week (**panel f**) old males. Glucose clearance after ip bolus of glucose (ipGTT) was significantly impaired at all time points for offspring of **maternal** diabetes, and at 30 and 60 minutes in 14 week old mice, and at 60 minutes after injection in 26 week old offspring of **paternal** diabetes. * Denotes statistically significant difference vs. control (*p<0.05, ** p<0.01, *** p<0.001).

Maternal diabetes was associated with lower body weight in male offspring compared to controls at 14 weeks. In the older mice, at 26 weeks of age, there was still a trend towards lower body weight compare to controls. (Figure 3A and Table 2) This difference in body weight developed with age, since weights of male offspring did not differ from controls until day 15 of life, when controls started to gain more weight. Longitudinal weight data from weaning to age 20 weeks are displayed in Figure 2D.

Maternal diabetes also resulted in significantly increased fasted glucose levels and of AUC, as a marker for glucose clearance after ip glucose challenge, in male wildtype offspring at 14 and 26 weeks of age (Figure 3B and 3C). In intraperitoneal glucose tolerance tests (ipGTT) the glucose clearance was significantly impaired (p<0.01) at all time points (baseline and 30, 60 and 120 minutes after injection) in 14 and 26 week old male offspring of maternal diabetes (Figure 3E and 3F and Table 2).

No significant differences between male offspring of diabetic mothers and controls were observed for serum levels of fasted insulin (Figure 3D), leptin or IGF-1. In accordance with the reduced body weight, males did show decreased lean body weight at 26 weeks, however percent body fat did not differ between offspring of diabetic mothers and controls (Table 2).

**Figure 4:**
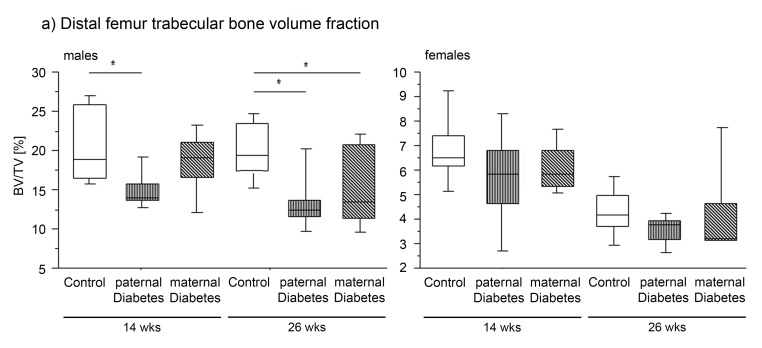
Percent of distal femur Bone Volume/Trabecular Volume (Tb.BV/TV (%)). Boxplots showing the smallest observation (lower bar), lower and upper quartile (box), median (line in box) and largest observation (upper bar) of male (left) and female (right) offspring of controls (open bars), paternal diabetes (longitudinal shading) and maternal diabetes (diagonal shading) for Percent of distal femur Bone Volume/Trabecular Volume (BV/TV %) at 14 and 26 weeks of age. * indicates p<0.05 vs. control.

Males had lower WBBMD and WBBMC compared to controls at 14 weeks of age. However by 26 weeks of age bone mineral density, content and area did not differ from controls any more (Table 2). While there were no significant differences in cortical bone properties, distal femur trabecular bone volume fraction (BV/TV, %) and trabecular thickness (Tb.Th) were lower in offspring of diabetic mothers compared to controls at 26 weeks (Figure 4).

#### Female offspring

Female offspring of maternal diabetes weighed less than controls at 14 and 26 weeks of age. (Table 3) Longitudinal weight data from weaning to age 20 weeks are displayed in Figure 2D.

Female mice also had disturbed glucose metabolism: the mice showed a trend toward elevated fasting glucose at 14 weeks, and a statistically significant elevation at 26 weeks of age. AUC was significantly increased at 14 weeks but less so at 26 weeks when the difference was no longer significant. Similar to the males, no significant changes in serum levels of fasted insulin and leptin were detected. In terms of body composition, significantly decreased lean body weight (LBW), body fat (BFat) and percent body fat developed by 26 weeks of age. (Table 3)

Female offspring of maternal diabetes showed no significant changes in bone properties at 14 weeks. However, at 26 weeks, WBBMD was significantly lower than in controls. In contrast to the males, there were no differences in trabecular bone microarchitecture in female offspring of diabetic mothers. Whereas in midshaft cortical bone, lower bone area fraction (BA/TA, %), bone area, and cortical thickness was observed at 26 weeks of age. These differences remained significant after body mass adjustment (BA/Cort.Th) (Figure 4 and Table 3)

### Effects of paternal diabetes on offspring

#### Male offspring

Wildtype male offspring of paternal diabetes weighed significantly less than controls at 14 and at 26 weeks of age. However, this reduction in body weight was less pronounced than the reduction seen in the offspring of maternal diabetes (Figure 3A and Table 2). Surprisingly, paternal diabetes resulted also in a significant length difference in the male offspring. Offspring of paternal diabetes was significantly longer at 14 weeks of age compared to controls (Table 2).

Fasted glucose levels (Figure 3B) did not differ in offspring of paternal diabetes at 14 weeks of age, but was elevated by 26 weeks of age. Additionally there was a trend towards increased AUC in the glucose tolerance tests (ipGTTs) at 14 and 26 weeks of age (Figure 3C). Glucose clearance in the ipGTTS was significantly impaired at 60 minutes (p<0.05) in 14 and in 26 week old male offspring of paternal diabetes (p<0.01 and <0.05 respectively) (Figure 3E and 3F).

Serum levels of fasted insulin did not differ from controls (Figure 3D). There was a trend toward lower leptin levels observed at 14 weeks. By 26 weeks of age leptin levels were significantly decreased in offspring of paternal diabetes (Table 2). No differences in Body composition were observed.

Wildtype male offspring of diabetic fathers developed a more severe skeletal phenotype than offspring of diabetic mothers. Skeletal changes affected bone mineral content, trabecular structure and cortical bone properties:

WBBMD was lower compared to controls at 14 weeks (Table 2). Distal femoral BV/TV and trabecular thickness were lower at 14 weeks of age and at 26 weeks (Figure 4), and number of trabeculae was lower at 14 weeks with a trend at 26 weeks (Table 2). In the midshaft femur, cortical bone area (BA) was lower at 14 weeks and cortical thickness was lower at both time points, both of which remained significant after body mass adjustment. (Table 2)

#### Female offspring

Female offspring of diabetic fathers were the least affected group in this study. At 14 weeks, of the properties measured, no significant differences from controls could be identified. At 26 weeks increased length in the female offspring of paternal diabetes was observed. Fasted glucose and AUC were not significantly different from controls, but there was a significant increase in lean body weight (LBW) at 26 weeks of age. (Table 3)

No differences in WBBMD, WBBMC or Bone Area were observed. Female offspring of diabetic fathers had a trend toward lower BV/TV at 14 weeks but otherwise did not differ from controls in their skeletal properties. (Figure 4 and Table 3)

### Summary

Metabolic parameters in wildtype offspring are affected more severely by maternal than paternal hyperglycemia. Conversely, skeletal development is affected more severely by paternal than maternal hyperglycemia. Furthermore, there is a strong sexual dimorphism in the response to parental hyperglycemia with male offspring demonstrating more metabolic and skeletal effects than female offspring. Impairment of metabolic physiology in offspring worsens over time and is more severe at 26 weeks, compared to 14 weeks of age, while impairment of skeletal physiology is more pronounced at the younger age.

## Discussion

Human epidemiological data point toward programming effects of diabetes and hyperglycemia on the developing organism; however, the mechanisms behind these effects are not well understood. Since an increasing number of pregnancies are affected by an altered metabolic environment due to maternal adipositas, high caloric input, gestational diabetes, or type 2 diabetes, it is of relevance to develop and investigate models that will help increase understanding of the mechanisms underlying programming of later in life diseases.

In recent years a variety of animal models of altered maternal metabolic environments due to overnutrition or diabetes have been developed. Researchers have used different types of high-fat or high sucrose diets (cafeteria diet), [15] [16] [17] [18], models of induced gestational diabetes [19], hyperglycemia via continuous glucose infusion [6] [8], streptozotocin induced diabetes models [18] [20] and other models of obesity and type 2 diabetes to investigate phenotypic outcome in offspring of these pregnancies.

From these studies, mostly rat and mouse models, we have learned that changes in the maternal environment can have lasting effects on offspring metabolic phenotypes. However, we have also learned that effects, effect sizes and timing of development of effects vary greatly. In some models a strong metabolic phenotype with the development of obesity can be observed, in other models the metabolic impairment is more subtle, affecting, for example, lipid metabolism. Besides metabolic impairments [15] [17], changes of skeletal development [16], of kidney function [21], of cardiac/vascular function [18], of aortic gene expression [20], of gene expression changes in hypothalamic centers [22], changes in pancreatic beta cells [23] and changes in behavior have been described as results of either diabetic or dysmetabolic mothers. More recently there have been reports on paternal transmission of such effects as well [16] [9].

Development and study of these models are important tools for attempting to tease apart the role of metabolic factors, hormone milieu, genetic background, and epigenetic changes in the development of offspring phenotypes after dysmetabolic/diabetic pregnancies. However, the mechanism has not yet been fully determined. With the Akita mouse we add an important addition to the armamentarium of models that can be used to study the effects of parental diabetes on offspring. In particular, this mouse allows for modeling of effects of maternal and paternal **insulin deficient** diabetes.

The combined data from our study demonstrate that offspring of parental diabetes develop metabolic and skeletal changes, whereas the particular phenotypic pattern and magnitude of abnormality depend on the sex and age of the offspring and whether the exposure was to maternal or paternal diabetes. This suggests that more than one mechanism might be involved in the programming of the phenotypes.

Uncovering the underlying mechanism(s) will contribute to our understanding of how parental environment can affect offspring phenotype. Thus, the molecular basis for the metabolic abnormalities in wildtype offspring of Akita parents an area of important future investigation. The impaired glucose tolerance may be the result of low insulin production, as shown in other models [19], [8] or of insulin resistance. To investigate this hypothesis further, we assessed fasted insulin levels. These were not significantly altered, but did trend towards lower levels in offspring of maternal diabetes than in controls, suggesting that insufficient insulin production may contribute to the phenotype.

Similar observations were made in a study by Gauguier et al (Gauguier 1991). The authors investigated insulin secretion in offspring of late pregnancy hyperglycemia in rats. The data indicated this metabolic perturbation resulted in impairment of insulin secretion and that the impairment may result from a perturbation of the neuroregulation of insulin secretion rather than an intrinsic pancreatic beta-cell defect. The importance of neuroregulation was also supported by data from Lau et al. indicating that a modulation of metabolic phenotype in offspring of diabetic mothers may stem from dysregulation of hypothalamic POMC and NPY gene expression in offspring. These authors postulate an additional effect of maternal hyperglycemia on genetic beta- cell dysfunction in offspring [19].

Alterations in offspring leptin levels are another possible underlying mechanism, but fasted leptin levels were not significantly altered by exposure to diabetes in Akita mice. This finding differs from data obtained from other models, such as *in-utero* overfeeding via obesogenic diets, where leptin resistance and increased weight gain have been observed [17]. Offspring in our model unexpectedly showed decreased bodyweight combined with a trend towards lower leptin levels. Since the amount of body fat in the experimental mice did not differ from controls, the apparent lack of leptin resistance may be one reason why offspring from our model did not experience increased weight gain.

Data from Lau et al [19] suggest that offspring phenotypes may be affected by degree of change in maternal environment. Heterozygous Akita mice usually develop hyperglycemia by 5–7 weeks of age [24], but the decline in beta-cell function may vary between individual Akita mice. We did not measure glucose levels in all our mice, and cannot exclude that levels of hyperglycemia varied between breeders. The subgroup of Akita mice that was tested before mating showed significantly elevated fasted glucose levels compared to controls. To further characterize the *intra-uterine* environment we sacrificed heterozygous Akita mothers and control breeders at day 17 of pregnancy and assessed maternal and fetal glucose levels. Our data indicate that, at least at this point in gestation, heterozygous and wildtype fetuses do not differ in their glucose levels. Exposure to in utero sibling hyperglycemia is a potential mechanism that could contribute to both the maternal and paternal effects we observed. However, our in utero data argue against a sibling in utero effect. This, of course, does not rule out other sibling effects caused by the Akita mutation.

Interestingly, not only maternal, but also paternal diabetes resulted in the development of metabolic changes in wildtype male offspring. Transmission of paternally inherited environmental information has been observed in other studies as well [9], [16], but mechanisms that underlie these effects are not well understood. The metabolic changes in offspring of paternal diabetes in our study were milder than the effects of maternal diabetes. A possible explanation for this different effect-size might be that maternal hyperglycemia results in direct programming of metabolic traits in the developing fetus, whereas the influence of paternal diabetes on offspring is indirect, possibly through transgenerationally transmitted marks of fetal programming from the father himself. This study was not designed to investigate effects of intra-uterine exposure to hyperglycemia of the parents of the breeders used, and thus was not powered sufficiently to detect effects on phenotypes that depend on the grandparental origin of diabetes. However, this area warrants further investigation in a larger scale experiment.

Quite surprisingly paternal diabetes resulted in mainly skeletal changes in male offspring. We were initially interested in skeletal data because of the report of Dunn et al showing a paternally transmitted length increase in 2^nd^ generation mice after the founding grandmaternal mice were fed a high fat diet as well as the effects of maternal high fat feeding on skeletal development [25]. We did, indeed, find a length difference with increased body length in male offspring of paternal diabetes but not for offspring of maternal diabetes. Furthermore we observed a decrease in overall bone mineral density and an impairment of trabecular properties in male offspring of paternal diabetes.

Changes in skeletal development in offspring as a result of maternal high-fat diet [25] and in offspring of rats with streptozotocin induced diabetes [21] have been described previously. The skeletal changes we observed could stem from parental transmission of programming effects, or be the result of the metabolic abnormalities in the offspring. Our data cannot differentiate these two possibilities fully, but they suggest that the bone phenotypes may not be secondary to the metabolic changes for the following reasons: (1) in offspring of maternal diabetes, skeletal changes are more pronounced in younger mice and improve with age, while the metabolic changes worsen; (2) in offspring of paternal diabetes, more severe skeletal changes develop in the presence of much milder metabolic changes.

An altered renal calcium and magnesium reabsorption was described in rat-offspring of streptozotocin induced diabetic dams [21] resulting in a decrease in trabecular bone and increase in cortical bone. We did not investigate calcium excretion with the urine, but it is possible that similar changes affect the kidneys in offspring of Akita mice. Future work should investigate Calcium-Bone physiology in these mice.

The same mechanism may underlie both the metabolic and skeletal phenotypes, or parental hyperglycemia may have altered multiple pathways that result in different phenotypes. One unifying hypothesis might be that the hypothalamus, as a regulator of energy metabolism and bone metabolism [26], is a target organ of fetal programming. The hypothalamus has been studied by other groups investigating fetal programming effects because of its significant role in energy metabolism. Changes in hypothalamic rat neurons after early overfeeding were found, indicating that the metabolism of these cells might be altered. A role for leptin in this regulation [22] was proposed, a thesis that has been supported by data from food restricted rats [27]. Interestingly differential methylation of relevant genes in hypothalamic samples from food-restricted animals has been described [22], pointing towards epigenetic modulation of hypothalamic areas, as a possible mechanism for fetal programming due to modified *intra-uterine* environments (Review: [23]).

Finally, we also observed a significant influence of offspring sex on the development of the phenotypes. While wildtype male offspring of maternal diabetes developed severe metabolic changes, findings in female offspring were mild. While most studies of fetal programming models focus on one sex in phenotypic evaluation of offspring, some have investigated both sexes. It has been shown previously that both parental origin and offspring sex play a role in transgenerational transmission of traits. However, the relevance of this in first generation offspring is unknown. [28] Additionally, some authors suggest a protective effect of estrogen for metabolic traits in females [29], a hypothesis that is not well understood in a fetal programming model and needs to be investigated further.

In summary the Akita mouse provides an important opportunity to investigate fetal programming effects in diabetes. It allows for future investigation of responsible mechanisms and may eventually allow for search of preventive interventions.

## Methods

### Ethics statement

All animal studies were approved by the Toronto Center for Phenogenomics Animal Care Committee (AUP 09-08-0097) in accordance with recommendations of the Canadian Council on Animal Care, the requirements under Animals for Research Act, RSO 1980, and the Toronto Centre for Phenogenomics (TCP) Committee Policies and Guidelines. Experimental mice were maintained under controlled conditions (25°C, 12-hour light/dark circle) at the TCP.

Akita mice (C57Bl6/J-Ins2 <Akita>, JAX #: 003548) carry a mutation in the Insulin-2 gene on the C57Bl6/J genetic background, which results in mis-folding of the insulin protein. Heterozygous mice are hyperglycemic by 5 weeks of age [10]. Akita mice were backcrossed to the 29^th^ generation on C57Bl6/J background as of January 2011. (For strain information see: http://jaxmice.jax.org/strain/003548.html)

For effects of **maternal** diabetes, heterozygous Akita females were bred with wildtype C57Bl6/J males and wildtype offspring were assessed. For effects of **paternal** hyperglycemia, heterozygous Akita males were bred with wildtype C57Bl6/J females, again wildtype offspring were assessed. Offspring of wildtype C57Bl6/J breeders, unrelated to the heterozygous Akita breeders, served as controls. Female Breeders were 6–12 weeks of age at time of breeding. For schematic representation of breeding strategy see Figure 1.

In a subgroup of animals, fasted maternal and paternal serum glucose concentrations were measured before mating, and on day 17 of pregnancy (females only) to ensure parental hyperglycemia in Akita animals. To assess a possible sibling effect via influence of genotype dependent fetal glucose levels on intra-uterine milieu, a subgroup of pregnant animals was sacrificed on day 17 of pregnancy and maternal and fetal glucose levels and genotypes were obtained. (Figure 2)

Males were removed after 3 days of breeding. Females were housed in pairs for 14 days and then moved to individual cages. Offspring of Akita breedings were kept with birth mothers and genotyped as described below on postnatal day 19–20. Wildtype offspring were selected and weaned into fresh cages with a maximum of 3 mice per cage.

Genotyping of all offspring from Akita matings was performed according to recommendations of Jackson Laboratory with the Ins2^Akita^ Restriction Reaction (Fnu4H1) and using Transnetyx core services (www.transnetyx.com). For detailed protocol see: “http://jaxmice.jax.org/protocolsdb/f?p=116:2:2757142631136909::NO:2:P2_MASTER_PROTOCOL_ID,P2_JRS_CODE:176,003548”

Intraperitoneal glucose tolerance tests (ipGTT) were performed in controls and wildtype offspring of Akita breedings at age 14 and 26 weeks. **ipGTTs** were performed after a 6-hour fast. A baseline fasted blood sample was obtained from the tail vein. Filtered, sterile Glucose (200 mg/ml) was injected ip at 2 g Glucose per kg bodyweight. Further samples were taken at 30, 60 and 120 minutes after the injection. Values for Area under the curve (AUC) of Glucose measurements at 0, 30, 60 and 120 minutes were calculated as: AUC = ½ ∑ (t_i+1_−t_i_) (y_i_+y_i+1_) where t = time point and y = measurement, according to Matthews [11].

Mice were sacrificed with CO2 inhalation after a 6-hour fast and terminal blood samples were obtained via cardiac puncture. Samples were kept on ice and spun at 20,000 rpm for 20 minutes to separate serum, before freezing at −20°C until further analysis. Length of offspring was measured postmortem in mm from nose to base of tail.

### Hormone Assays and Glucose measurements

Serum insulin was measured using Insulin (Mouse) Ultrasensitive EIA Kit (ALPCO Diagnostics, NH, USA), leptin using Mouse Leptin ELISA Kit (Crystal Chem Inc. IL, USA), IGF-1 using Mouse IGF-1 RIA, (ALPCO, Windham, NH, USA). Blood glucose was measured using the BAYER Contour Glucometer.

### Bone mineral density and body composition

Whole body (excluding head region) bone mineral density (WBBMD, g/cm2), bone mineral content (WBBMC, g), bone area (cm^2^), lean mass (g), fat mass (g), and body composition (% body fat) were measured following euthanasia using peripheral dual-energy X-ray absorptiometry (pDXA, PIXImusII, GE Lunar Corp., Madison, WI) [12].

### Trabecular and cortical bone morphology by μCT

The right femur was dissected, wrapped in saline-soaked gauze and frozen at −20°C. Cortical and trabecular bone morphology and microarchitecture were measured using high-resolution μCT (μCT40, Scanco Medical, Brüttisellen, Switzerland) [13]. Briefly, the distal femoral metaphysis was scanned using an X-ray energy of 70 KeV, integration time of 200 ms, and 12 µm isotropic voxel sizes. For distal femoral trabecular bone region, bone volume fraction (BV/TV, %), trabecular thickness (Tb.Th, µm), trabecular separation (Tb.Sp, µm), trabecular number (Tb.N, 1/mm), connectivity density (ConnD, 1/mm^3^), and structure model index (SMI) were assessed. For midshaft cortical bone region, transverse CT slices were acquired to measure total cross-sectional area, cortical bone area and medullary area (TA, BA and MA, mm2); cortical bone area fraction (BA/TA, %), cortical thickness (mm), and area moments of inertia (maximum, Imax; minimum, Imin; and polar, pMOI; mm4) [14].

### Numbers of Animals included in the studies

A total of 322 mice were phenotyped and included in the analyses. Of these length data was obtained in 140, fasted glucose in 283, fasted insulin in 129, fasted leptin in 80 and IGF-1 in 42. IpGTTs were performed in 261, pDXA scans in 131 and μCTs in 134.

### Statistical analysis of phenotypic data

Results are given as mean ± SD or as mean with 95% confidence interval, as indicated. Statistical significance was attributed to *P*<0.05, after correction for multiple testing. Continuous variables were analysed for the effects of maternal/paternal diabetes, age and sex using three-factorial ANOVA (Proc GLM), including the effect of interaction between factors. Further, effects of maternal and paternal diabetes were analysed separately for each sex and age group using ANOVA, followed by Tukey's test for differences between groups. Analysis was performed using the SAS™ software (SAS Corporation, Carey NJ, USA).

## Supporting Information

Table S1 **Complete results of phenotypic markers in offspring of maternal diabetes, paternal diabetes and controls.** Mean ± STD and animal numbers are provided for **male (Table S1A) and female (Table S1B)** offspring at 14 and 26 weeks of age for all phenotypic markers obtained in offspring of maternal diabetes, paternal diabetes and controls. Significant differences to control offspring (p<0.05) are highlighted in grey shaded boxes (* p<0.05, ANOVA). AUC: Area under the curve for Glucose response after ipGTT, BMD: Bone Mineral Density, BMC: Bone Mineral Content, LBW: Lean Body Weight, BFAT: Body Fat, Tb. Th.: trabecular thickness, Tb.N.: number of trabeculae, Tb. Sp.: trabecular separation, Conn Dens: connectivity density, SMI: structure model index, Cort. Area: Cortical area , BA/TA %: Bone Area/Trabecular Area, Cort. Th.: Cortical thickness , Imax: maximum moment of inertia, Imin: minimum moment of inertia, pMOI: polar moment of inertia
(DOC)
