# Supplementary material for: Parental Diabetes: The Akita Mouse as a Model of the Effects of Maternal and Paternal Hyperglycemia in Wildtype Offspring
Source: PLoS One. 2012 Nov 28;7(11):e50210. doi: 10.1371/journal.pone.0050210 (PMC3509145; doi:10.1371/journal.pone.0050210)
Supplement: Table S1 — Complete results of phenotypic markers in offspring of maternal diabetes, paternal diabetes and controls. Mean ± STD and animal numbers are provided for male (Table S1A) and female (Table S1B) offspring at 14 and 26 weeks of age for all phenotypic markers obtained in offspring of maternal diabetes, paternal diabetes and controls. Significant differences to control offspring (p<0.05) are highlighted in grey shaded boxes (* p<0.05, ANOVA). AUC: Area under the curve for Glucose response after ipGTT, BMD: Bone Mineral Density, BMC: Bone Mineral Content, LBW: Lean Body Weight, BFAT: Body Fat, Tb. Th.: trabecular thickness, Tb.N.: number of trabeculae, Tb. Sp.: trabecular separation, Conn Dens: connectivity density, SMI: structure model index, Cort. Area: Cortical area , BA/TA %: Bone Area/Trabecular Area, Cort. Th.: Cortical thickness , Imax: maximum moment of inertia, Imin: minimum moment of inertia, pMOI: polar moment of inertia (DOC) [file pone.0050210.s001.doc]

| **Supplementary Table S1a) Male Offspring** | | | | | | | | | | | |
| --- | --- | --- | --- | --- | --- | --- | --- | --- | --- | --- | --- |
|  | | | **Maternal Diabetes** | | | **Paternal Diabetes** | | | **Controls** | | |
|  | | **Unit** | **Mean** | **STD** | **n** | **Mean** | **STD** | **n** | **Mean** | **STD** | **n** |
| **14 weeks** | Weight | g | **25.45 *** | **2.37** | **35** | **26.02 *** | **1.65** | **29** | 27.6 | 1.74 | 62 |
|  | Length | cm | 9.75 | 0.21 | 16 | **10.1 *** | **0.2** | **5** | 9.81 | 0.17 | 20 |
|  | Glucose | mg/dl | **9.64 *** | **1.67** | **33** | 8.6 | 1.5 | 26 | 8.52 | 1.69 | 53 |
|  | AUC |  | **2279 *** | **672.7** | **32** | 1984.13 | 514.9 | 23 | 1692.8 | 527.6 | 47 |
|  | Insulin | ng/ml | 0.98 | 0.31 | 13 | 0.98 | 0.41 | 7 | 1.23 | 0.56 | 17 |
|  | Leptin | ng/ml | 1.14 | 0.65 | 9 | 1.66 | 1.78 | 6 | 2.4 | 1.8 | 10 |
|  | IGF-1 | ng/ml | 239.8 | 32.3 | 9 | 245.6 | 13.8 | 5 | 252.2 | 38.8 | 10 |
|  | BMD | g/cm^2^ | **0.05 *** | **0.003** | **16** | **0.049 *** | **0.003** | **5** | 0.053 | 0.0019 | 20 |
|  | BMC | g | **0.43 *** | **0.052** | **16** | 0.42 | 0.039 | 5 | 0.48 | 0.037 | 20 |
|  | Area | cm^2^ | **8.45 *** | **0.55** | **16** | 8.66 | 0.353 | 5 | 9.04 | 0.49 | 20 |
|  | LBW | g | 20.4 | 1.6 | 16 | 20.35 | 1.26 | 5 | 21.73 | 1.72 | 20 |
|  | BFAT | g | 4.83 | 1.07 | 16 | 4.8 | 0.61 | 5 | 5.19 | 0.74 | 20 |
|  | %Fat |  | 18.9 | 2.9 | 16 | 18.98 | 2.25 | 5 | 19.37 | 3.28 | 20 |
|  | Tb.Th | mm | 0.057 | 0.007 | 8 | **0.052 *** | **0.006** | **7** | 0.058 | 0.006 | 17 |
|  | Tb.N | /mm | 4.96 | 0.36 | 8 | **4.72 *** | **0.15** | **7** | 5.1 | 0.39 | 17 |
|  | Tb.Sp | mm | 0.191 | 0.014 | 8 | 0.203 | 0.007 | 7 | 0.184 | 0.016 | 17 |
|  | Conn Dens | mm^-3^ | 132.7 | 33.37 | 8 | 121.67 | 13.46 | 7 | 149.06 | 27.72 | 17 |
|  | SMI |  | 2.08 | 0.43 | 8 | 2.41 | 0.29 | 7 | 1.9 | 0.55 | 17 |
|  | Cort. area | mm^2^ | 0.808 | 0.123 | 8 | **0.749 *** | **0.071** | **8** | 0.818 | 0.083 | 17 |
|  | Total area | mm^2^ | 1.86 | 0.294 | 8 | 1.806 | 0.163 | 8 | 1.903 | 0.175 | 17 |
|  | BA/TA | % | 43.5 | 1.7 | 8 | 41.5 | 1.6 | 8 | 43 | 2.5 | 17 |
|  | Cort. Th. | mm | 0.178 | 0.012 | 8 | **0.168 *** | **0.009** | **8** | 0.177 | 0.01 | 17 |
|  | Imax | mm^4^ | 0.279 | 0.081 | 8 | 0.261 | 0.052 | 7 | 0.284 | 0.048 | 17 |
|  | Imin | mm^4^ | 0.13 | 0.037 | 8 | 0.113 | 0.021 | 8 | 0.133 | 0.029 | 17 |
|  | pMOI | mm^4^ | 0.409 | 0.117 | 8 | 0.374 | 0.072 | 7 | 0.418 | 0.074 | 17 |
| **26 weeks** | Weight | g | 30.4 | 1.9 | 18 | **29.8 *** | **1.8** | **18** | 31.9 | 2.56 | 38 |
|  | Length | cm | 10.1 | 0.58 | 7 | 10.25 | 0.26 | 10 | 10.2 | 0.29 | 20 |
|  | Glucose | mmol/l | **9.01 *** | **1.74** | **17** | **9.03 *** | **2.19** | **18** | 7.4 | 1.66 | 38 |
|  | AUC |  | **2425 *** | **797** | **15** | 1799.2 | 512 | 18 | 1575 | 418 | 37 |
|  | Insulin | ng/ml | 0.76 | 0.24 | 8 | 1.1 | 0.54 | 6 | 1.23 | 0.87 | 34 |
|  | Leptin | ng/ml | 1.78 | 0.38 | 6 | **0.56 *** | **0.43** | **4** | 3.63 | 2.2 | 19 |
|  | BMD | g/cm^2^ | 0.053 | 0.003 | 7 | 0.055 | 0.003 | 10 | 0.056 | 0.003 | 20 |
|  | BMC | g | 0.48 | 0.06 | 7 | 0.48 | 0.044 | 10 | 0.51 | 0.039 | 20 |
|  | Area | cm^2^ | 9.05 | 0.6 | 7 | 8.81 | 0.78 | 10 | 9.16 | 0.48 | 20 |
|  | LBW | g | **22.8 *** | **1.6** | **7** | 23.7 | 1.6 | 10 | 24.99 | 1.84 | 20 |
|  | BFAT | g | 6.02 | 1.1 | 7 | 5.98 | 1.9 | 10 | 5.93 | 1.1 | 20 |
|  | %Fat |  | 20.7 | 3.35 | 7 | 19.81 | 4.2 | 10 | 19.1 | 2.9 | 20 |
|  | Tb.Th | mm | **0.058 *** | **0.006** | **8** | **0.059 *** | **0.006** | **7** | 0.065 | 0.004 | 18 |
|  | Tb.N | /mm | 4.23 | 0.16 | 8 | 4.1 | 0.32 | 7 | 4.34 | 0.34 | 18 |
|  | Tb.Sp | mm | 0.223 | 0.011 | 8 | 0.232 | 0.021 | 7 | 0.213 | 0.021 | 18 |
|  | Conn Dens | mm^-3^ | 79.51 | 12.46 | 8 | 69.97 | 22.29 | 7 | 91.37 | 26.14 | 18 |
|  | SMI |  | 2.32 | 0.75 | 8 | 2.57 | 0.65 | 7 | 1.88 | 0.51 | 18 |
|  | Cort. area | mm^2^ | 0.84 | 0.088 | 8 | 0.794 | 0.052 | 7 | 0.877 | 0.064 | 18 |
|  | Total area | mm^2^ | 1.938 | 0.288 | 8 | 2.022 | 0.269 | 7 | 2.085 | 0.223 | 18 |
|  | BA/TA | % | 43.7 | 3.7 | 8 | 39.6 | 3 | 7 | 42.3 | 3.1 | 18 |
|  | Cort. Th. | mm | 0.178 | 0.01 | 8 | **0.164 *** | **0.008** | **7** | 0.179 | 0.011 | 18 |
|  | Imax | mm^4^ | 0.306 | 0.07 | 8 | 0.303 | 0.037 | 7 | 0.331 | 0.057 | 18 |
|  | Imin | mm^4^ | 0.137 | 0.038 | 8 | 0.141 | 0.043 | 7 | 0.158 | 0.03 | 18 |
|  | pMOI | mm^4^ | 0.442 | 0.106 | 8 | 0.444 | 0.079 | 7 | 0.489 | 0.084 | 18 |

|  | **Supplementary Table S1b) Female Offspring** | | | | | | | | | | |
| --- | --- | --- | --- | --- | --- | --- | --- | --- | --- | --- | --- |
|  |  |  | **Maternal Diabetes** | | | **Paternal Diabetes** | | | **Controls** | | |
|  |  | **Unit** | **Mean** | **STD** | **n** | **Mean** | **STD** | **n** | **Mean** | **STD** | **n** |
| **14 weeks** | Weight | g | **19.22 *** | **0.97** | **18** | 20.48 | 1.42 | 27 | 20.69 | 1 | 42 |
|  | Length | cm | 9.4 | 0.3 | 3 | 9.31 | 0.21 | 16 | 9.15 | 0.27 | 16 |
|  | Glucose | mmol/l | 8.46 | 1.65 | 14 | 7.46 | 0.95 | 6 | 7.46 | 1.33 | 36 |
|  | AUC |  | **1932.8 *** | **787.2** | **14** | 1700.2 | 688.8 | 6 | 1405.47 | 458.9 | 35 |
|  | Insulin | ng/ml | 0.43 | 0.19 | 8 | na | na |  | 0.42 | 0.64 | 25 |
|  | Leptin | ng/ml | 1.26 | 0.24 | 8 | na | na |  | 0.77 | 0.27 | 7 |
|  | BMD | g/cm^2^ | 0.046 | 0.001 | 3 | 0.048 | 0.002 | 5 | 0.049 | 0.002 | 16 |
|  | BMC | g | 0.39 | 0.031 | 3 | 0.4 | 0.03 | 5 | 0.44 | 0.053 | 16 |
|  | Area | cm^2^ | 8.42 | 0.39 | 3 | 8.37 | 0.3 | 5 | 8.97 | 0.77 | 16 |
|  | LBW | g | 16.27 | 1.3 | 3 | 16.74 | 1.36 | 5 | 15.94 | 1.19 | 16 |
|  | BFAT | g | 3.76 | 0.47 | 3 | 4.18 | 0.43 | 5 | 3.75 | 0.55 | 16 |
|  | %Fat |  | 18.66 | 1.05 | 3 | 19.9 | 0.67 | 5 | 19.12 | 3.03 | 16 |
|  | Tb.Th | mm | 0.05 | 0.005 | 8 | 0.047 | 0.004 | 6 | 0.05 | 0.005 | 19 |
|  | Tb.N | /mm | 3.41 | 0.21 | 8 | 3.38 | 0.5 | 6 | 3.5 | 0.27 | 19 |
|  | Tb.Sp | mm | 0.294 | 0.021 | 8 | 0.299 | 0.053 | 6 | 0.285 | 0.023 | 19 |
|  | Conn Dens | mm^-3^ | 29.28 | 15.38 | 8 | 30.47 | 22.02 | 6 | 32.78 | 11.9 | 19 |
|  | SMI |  | 3.48 | 0.32 | 8 | 3.44 | 0.23 | 6 | 3.38 | 0.19 | 19 |
|  | Cort. area | mm^2^ | 0.68 | 0.05 | 8 | 0.699 | 0.101 | 6 | 0.702 | 0.039 | 20 |
|  | Total area | mm^2^ | 1.566 | 0.103 | 8 | 1.576 | 0.137 | 6 | 1.577 | 0.066 | 20 |
|  | BA/TA | % | 43.5 | 2.4 | 8 | 44.2 | 2.9 | 6 | 44.5 | 1.4 | 20 |
|  | Cort. Th. | mm | 0.169 | 0.011 | 8 | 0.173 | 0.019 | 6 | 0.175 | 0.007 | 20 |
|  | Imax | mm^4^ | 0.187 | 0.025 | 8 | 0.195 | 0.047 | 6 | 0.192 | 0.022 | 19 |
|  | Imin | mm^4^ | 0.094 | 0.011 | 8 | 0.097 | 0.019 | 6 | 0.097 | 0.008 | 20 |
|  | pMOI | mm^4^ | 0.281 | 0.035 | 8 | 0.292 | 0.064 | 6 | 0.29 | 0.028 | 19 |
| **26 weeks** | Weight | g | **21.05 *** | **1.35** | **7** | 24.98 | 2.11 | 15 | 24.16 | 1.63 | 28 |
|  | Length | cm | 9.48 | 0.21 | 5 | **10.13 *** | **0.23** | **9** | 9.73 | 0.28 | 20 |
|  | Glucose | mmol/l | **10.01 *** | **1.16** | **6** | 8.43 | 1.22 | 12 | 7.97 | 1.77 | 24 |
|  | AUC |  | 1614.5 | 210.1 | 6 | 1370.7 | 325.3 | 5 | 1574.75 | 437.3 | 24 |
|  | Insulin | ng/ml | 0.39 | 0.07 | 5 | na | na |  | 0.52 | 0.16 | 12 |
|  | Leptin | ng/ml | 0.94 | 0.06 | 5 | na | na |  | 1.66 | 0.91 | 10 |
|  | BMD | g/cm^2^ | **0.052 *** | **0.001** | **7** | 0.055 | 0.002 | 9 | 0.055 | 0.001 | 20 |
|  | BMC | g | 0.46 | 0.03 | 7 | 0.47 | 0.03 | 9 | 0.48 | 0.02 | 20 |
|  | Area | cm^2^ | 8.95 | 0.54 | 7 | 8.58 | 0.44 | 9 | 8.84 | 0.41 | 20 |
|  | LBW | g | **16.84 *** | **1.15** | **7** | **19.96 *** | **1.06** | 9 | 18.66 | 1.37 | 20 |
|  | BFAT | g | **3.71 *** | **0.35** | **7** | 5.62 | 1.24 | 9 | 5.19 | 1.01 | 20 |
|  | %Fat |  | **18.05 *** | **1.45** | **7** | 21.85 | 3.67 | 9 | 21.64 | 2.98 | 20 |
|  | Tb.Th | mm | 0.048 | 0.006 | 7 | 0.05 | 0.006 | 9 | 0.05 | 0.005 | 18 |
|  | Tb.N | /mm | 2.76 | 0.14 | 7 | 2.61 | 0.3 | 9 | 2.66 | 0.19 | 18 |
|  | Tb.Sp | mm | 0.362 | 0.02 | 7 | 0.387 | 0.054 | 9 | 0.373 | 0.028 | 18 |
|  | Conn Dens | mm^-3^ | 17.14 | 12.82 | 7 | 13.72 | 6.8 | 9 | 20.17 | 7.2 | 18 |
|  | SMI |  | 3.4 | 0.43 | 7 | 3.58 | 0.23 | 9 | 3.37 | 0.18 | 18 |
|  | Cort. area | mm^2^ | **0.761 *** | **0.043** | **7** | 0.84 | 0.033 | 9 | 0.836 | 0.044 | 18 |
|  | Total area | mm^2^ | 1.601 | 0.104 | 7 | 1.723 | 0.075 | 9 | 1.699 | 0.101 | 18 |
|  | BA/TA | % | **47.6 *** | **2** | **7** | 48.8 | 1.3 | 9 | 49.2 | 1.8 | 18 |
|  | Cort. Th. | mm | **0.192 *** | **0.009** | **7** | 0.203 | 0.006 | 9 | 0.203 | 0.007 | 18 |
|  | Imax | mm^4^ | 0.202 | 0.022 | 7 | 0.245 | 0.021 | 9 | 0.243 | 0.036 | 18 |
|  | Imin | mm^4^ | 0.107 | 0.015 | 7 | 0.122 | 0.01 | 9 | 0.119 | 0.012 | 18 |
|  | pMOI | mm^4^ | 0.309 | 0.035 | 7 | 0.367 | 0.03 | 9 | 0.362 | 0.045 | 18 |
